# Characterization of artificially re-pigmented ARPE-19 retinal pigment epithelial cell model

**DOI:** 10.1038/s41598-019-50324-8

**Published:** 2019-09-24

**Authors:** Laura Hellinen, Marja Hagström, Heidi Knuutila, Marika Ruponen, Arto Urtti, Mika Reinisalo

**Affiliations:** 1 School of Pharmacy, Faculty of Health Sciences, University of Eastern Finland, 70210 Kuopio, Finland; 2 Drug Research Programme, Division of Pharmaceutical Biosciences, Faculty of Pharmacy, University of Helsinki, P.O. Box 56, FI-00014 Helsinki, Finland; 3 Laboratory of Biohybrid Technologies, Institute of Chemistry, St. Petersburg State University, Peterhoff, 198504 St Petersburg, Russian Federation Russia; 4 Institute of Clinical Medicine, Department of Ophthalmology, Faculty of Health Sciences, University of Eastern Finland, 70210 Kuopio, Finland

**Keywords:** Drug delivery, Multivesicular bodies

## Abstract

Melanin pigment has a significant role in ocular pharmacokinetics, because many drugs bind at high extent to melanin in the retinal pigment epithelial cells. Most retinal pigment epithelial cell lines lack pigmentation and, therefore, we re-pigmented human ARPE-19 cells to generate a pigmented cell model. Melanosomes from porcine retinal pigment epithelium were isolated and co-incubated with ARPE-19 cells that spontaneously phagocytosed the melanosomes. Internalized melanosomes were functionally integrated to the cellular system as evidenced by correct translocation of cellular Rab27a protein to the melanosomal membranes. The pigmentation was retained during cell cultivation and the level of pigmentation can be controlled by altering the amount of administered melanosomes. We used these cells to study melanosomal uptake of six drugs. The uptake was negligible with low melanin-binders (methotrexate, diclofenac) whereas most of the high melanin-binders (propranolol, chloroquine) were extensively taken up by the melanosomes. This cell line can be used to model pigmentation of the retinal pigment epithelium, while maintaining the beneficial cell line characteristics, such as fast generation of cultures, low cost, long-term maintenance and good reproducibility. The model enables studies at normal and decreased levels of pigmentation to model different retinal conditions.

## Introduction

Retinal pigment epithelium (RPE) is a heavily pigmented cell monolayer between the neural retina and highly vascularized choroid. The RPE is essential for the vision, since it maintains photoreceptor viability and functionality by phagocytosing shed photoreceptor outer segments^1^. The RPE also maintains retinoid cycle for phototransduction and protects the retina against solar radiation^2^. Melanin pigment is synthesized in the RPE cells and packed in melanosomes that are pigmented cell organelles^3^. Most natural melanins are a mixture of brown/black eumelanin and yellow/red pheomelanin^4^. The RPE melanosomes contain mainly eumelanin^5,6^ whereas melanocytes of iris and choroid display varying eumelanin/pheomelanin ratios depending on the iris color^7^. Melanin protects the eye and skin^4^ against sunlight (including ultraviolet radiation)^5,6^, scavenges free radicals and maintains metal ion homeostasis in the retina^7^. Melanin is also present in various other tissues in the human body (e.g. inner ear and brain). Some disorders cause alterations in the levels of melanin in skin and eye (e.g. forms of oculocutaneous albinism)^8^, whereas other disorders (e.g. ocular albinism, retinitis pigmentosa, age-related macular degeneration) specifically reduce the pigmentation in the RPE and other ocular tissues^9^. Pathological conditions associated with altered synthesis of melanin and melanosomes can also affect the homeostasis of the eye and visual acuity.

The RPE forms outer blood-retinal barrier that limits the access of drugs and other xenobiotics from the systemic blood stream into the eye. In addition, the RPE is damaged in many ocular diseases (e.g. geographic atrophy) that cause impaired vision^10^. Vision threatening diseases, such as age-related macular degeneration, become more common in the aging populations and new drug treatments are needed. The RPE is certainly an important drug target and pharmacokinetic barrier.

Melanin affects ocular pharmacokinetics, because many drugs bind and accumulate to melanin^11–16^. Recent study of >3000 compounds demonstrated that melanin binding is a common feature among drug-like compounds^17^. Binding of drugs to melanin can lead to prolonged drug action in pigmented ocular tissues^18^, reduced peak responses of drugs^19^ and drug accumulation into the pigmented ocular tissues^20^. Overall, the pharmacokinetic consequences of melanin binding are a result from a complex interplay between melanin binding and drug permeability in the cells^16^. It is important to understand pigment related pharmacokinetics at cellular level to find optimal compounds with prolonged drug action in the retina. Prolongation of drug action and dosing intervals are needed in the retinal treatments, because small molecular drugs are otherwise rapidly eliminated after intravitreal injections^21^.

Lack of proper RPE cell lines hampers development of ocular drugs. Several continuous RPE cell lines are commercially available, but they tend to lose the original pigmented phenotype of the RPE^16,22^. Human RPE cell line ARPE-19^23^ is widely used to study retinal cell biology, pathological conditions and pharmacology, but ARPE-19 cells lack melanin pigment. Otherwise, ARPE-19 cell line shows appropriate features, such as barrier formation and expression of transporters^24,25^. Considering the pharmacokinetic impact and other functions of melanin in the RPE cells, non-pigmented cells are sub-optimal models of the RPE^22,26^.

Unfortunately, cultures of pigmented primary RPE cells and pigmented stem cell-derived RPE cells are poorly reproducible and they require time consuming steps of cell isolation and culture^22,27^. Therefore, easy-to-use RPE-like cell lines would be beneficial in retinal research and drug discovery. Chemicals or optimized culture conditions have been used to induce cellular pigmentation^22,28^, but utilization of phagocytosis of the RPE cells seems to be the most efficient and the fastest technique to re-pigment RPE cells. Feeding of melanin or melanosomes from different sources have been used to re-pigment depigmented cells^7,29–32^. Nevertheless, viability and membrane integrity of the isolated melanosomes have not been studied and, in some cases, the melanosomal membrane has been removed before feeding into the cells^32^. The lack of functional melanosomal membrane compromises important melanosomal functions (e.g. ion transport)^33,34^. Furthermore, intact melanosomal membrane is expected to extend the retention of pigment bound drug depot in the cells^16^.

In this study, we generated artificially pigmented RPE cell model (ARPE-19mel) from commonly used ARPE-19 cell line by feeding them with isolated porcine RPE melanosomes. Our melanosome isolation technique produces pure fractions of intact and functional melanosomes with ATPase activity^34^. Here, we studied the fate of melanosomes in the host cells and demonstrated that the melanosomal membrane remains intact after their isolation and cellular internalization. Importantly, physiologically relevant pigmentation in the RPE cells was achieved. In addition, we determined melanosomal uptake of six drugs that have different affinities to melanin. Our cell model enables quantitative control of melanosome quantity in the cells and provides a valuable *in vitro* tool for controlled pharmacological RPE studies.

## Results

### Melanosome content in ARPE-19mel cells can be controlled

**Figure 1:**
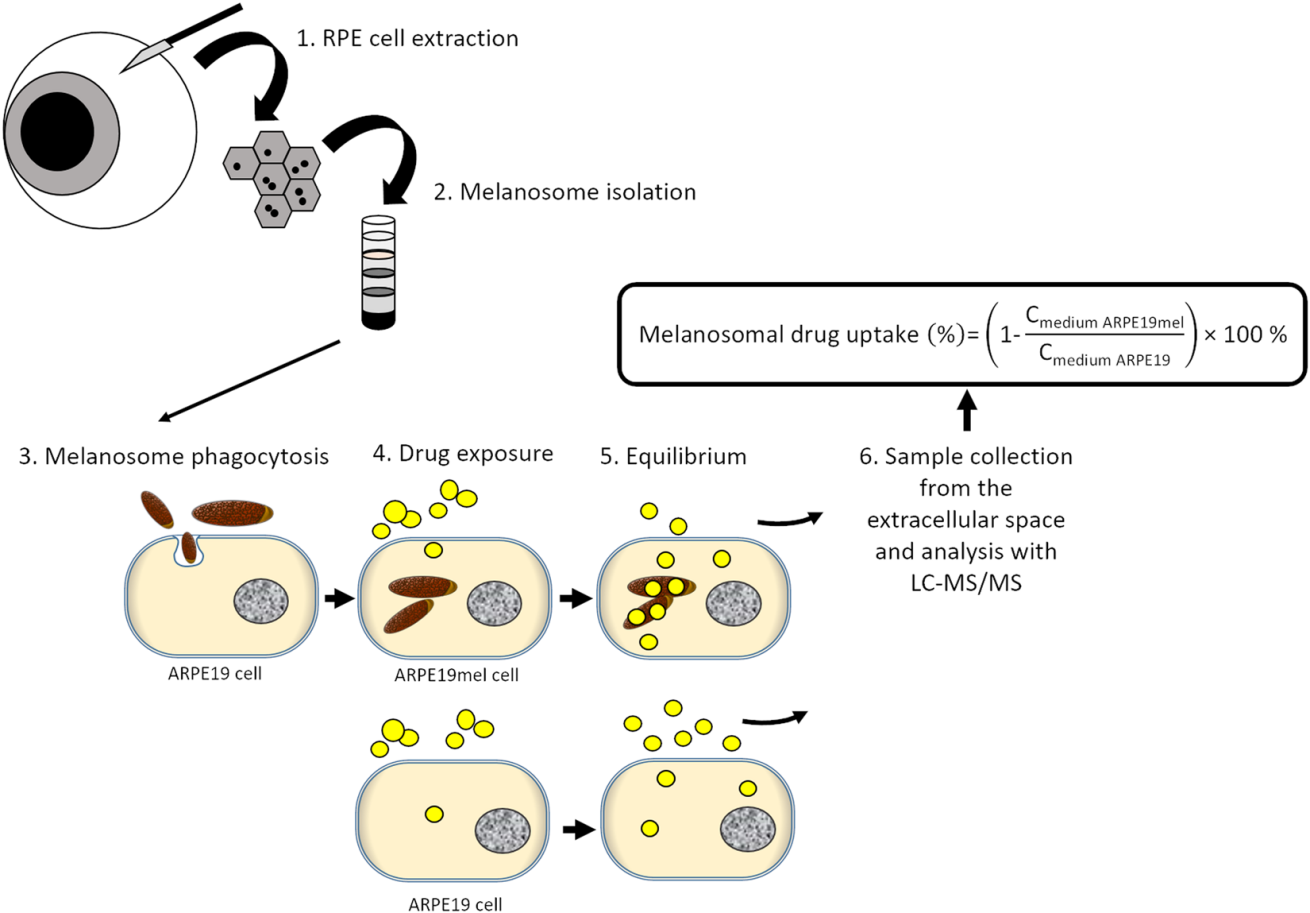
Schematic presentation of the ARPE-19mel cell model generation and the melanosomal drug uptake assay protocol.

In this work, we generated pigmented ARPE-19mel cells by administering isolated porcine melanosomes into regular ARPE-19 cell cultures using the method shown in Fig. 1.

**Figure 2:**
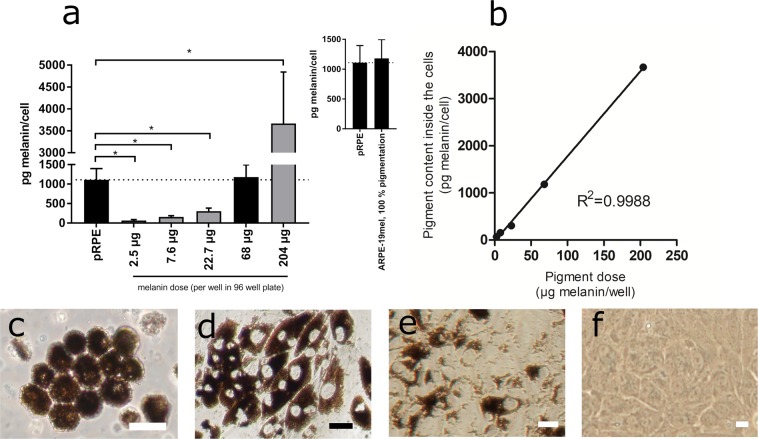
ARPE-19mel cell model characterization revealed optimal conditions for obtaining physiologically relevant stage of pigmentation (**a**). Melanin content in ARPE-19mel cells 6 days after melanosome dosing. The bars display mean values and error bars show standard deviations (SD). Melanosome dose corresponding to 68 µg melanin (n = 9) resulted in equal cellular pigmentation as the porcine RPE (n = 8). Other conditions resulted in different levels of cellular pigmentation as compared to the porcine RPE (*p < 0.001, n = 6 in each condition). The statistical significance was determined with un-paired t-tests using FDR approach (two-stage linear step-up procedure of Benjamini, Krieger and Yekutieli, with Q = 1%, without assuming a consistent SD). The inset displays porcine RPE (pRPE) and ARPE-19mel pigmentation levels for the cells that were exposed to 68 µg of melanin per well (i.e. marked as 100% pigmentation levels). (**b**) Linear correlation between exposed melanin amount and cellular pigmentation. Non-pigmented ARPE-19 cells (**f**) phagocytose isolated porcine RPE melanosomes. Melanosomal exposure of the ARPE-19 cells is used to control the pigmentation of the ARPE-19mel cells from moderate (**e**) to heavy pigmentation (**d**). In ARPE-19mel cells, level of pigmentation can be adjusted similar (**d**) to normal pigmentation of the porcine RPE (**c**). The cell images (**c**–**f**) were obtained with regular light microscope. Scale bars: (**c**) 20 µm, (**d**) 50 µm, (**e**) 20 µm, (**f**) 20 µm.

Six days after melanosome administration, dose-dependent levels of melanosomes in the ARPE-19mel cells were evident (Fig. 2a,b,d–f). Linear correlation between the melanosome dose and resulting melanin amount in the cells was demonstrated (Fig. 2b, R^2^ = 0.9988). This indicates that the melanin content inside the cells can be conveniently controlled. The required pigment dose can be estimated with linear regression equation (y = 18.025x  −22.84) when the desired pigment content is known (Fig. 2b). The equation can be used if the cells are cultured with the same procedures as in this study.

We quantitated the melanin content in the ARPE-19mel cells after culturing them on 96-well plates for 6 days after exposing the cells to melanosomes (2.5–204 µg melanin/well) (Fig. 2a). The melanin contents of ARPE-19mel cells were compared with native, non-cultured porcine RPE (pRPE, Fig. 2a). At the highest melanosome dose (204 µg melanin/well), the cellular pigment reached 3700 pg melanin/cell, which is higher than the pigment content in the porcine RPE (1110 pg melanin/cell, Fig. 2a). However, when the highest melanosome dose (204 µg/well) was loaded into the cells three times, no further increase in cellular pigmentation was observed (data not shown). Delivering a melanin dose of 68 µg/well to the ARPE-19 cells resulted in equivalent melanin content (1180 pg/cell) with freshly isolated porcine RPE cells (1110 pg/cell) (Fig. 2a, Supplementary Table 1). The other pigment doses resulted in different levels of cellular melanin (statistical significance: 2.5 µg p = 0.0000014, n = 6; 7.6 µg p = 0.0000035, n = 6; 22.7 µg p = 0.000024, n = 6; 204 µg p = 0.000025, n = 6). The physiologically relevant pigment content was retained in the cells for the culture time (6–7 days), as their calculated pigment recovery was approximately 102% (with a range of 63–127%, Supplementary Table 1). Detailed data of the pigment content per cell and pigment recoveries are presented in Supplementary material.

Hereafter, we describe the ARPE-19mel cells that were generated with 68 µg melanin/ well. These cells had similar melanin content as the primary porcine RPE cells (i.e. marked as 100% pigmentation) (Fig. 2a).

### Rab27a translocated into melanosomal membranes

**Figure 3:**
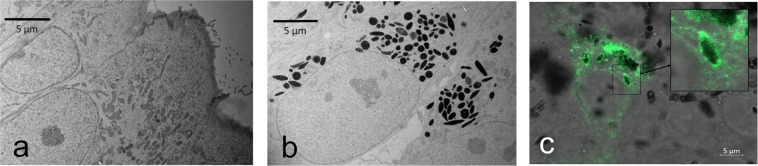
Melanosomal membrane remains intact during the organelle feeding and internalization to ARPE-19 cells. Transmission electron microscopy images demonstrate cytoplasmic localization of the melanosomes in re-pigmented ARPE-19mel cells (**b**), but not in normal ARPE-19 cells (**a**) (both images display 2000x magnification and 5 µm scale bars). Endogenously expressed melanosome protein Rab27a in ARPE-19mel cells localizes in the membranes of the transferred melanosomes (5 µm scale bar) (**c**). The inset shows single melanosome with enriched GFP-Rab27a protein in melanosome membrane two days after transfection.

Intracellular localization of melanosomes in the cytoplasm of ARPE-19mel cells was seen with transmission electron microscopy (Fig. 3b). No pigmentation was seen in the normal ARPE-19 cells (Fig. 3a). After transfection of pigmented ARPE-19mel cells with GFP-Rab27a vector, expression and cellular localization of GFP-Rab27a fusion protein was followed by using confocal microscopy. Rab27a was localized in the melanosome membrane (Fig. 3c).

### Melanin binding of drugs in ARPE-19mel cells

**Figure 4:**
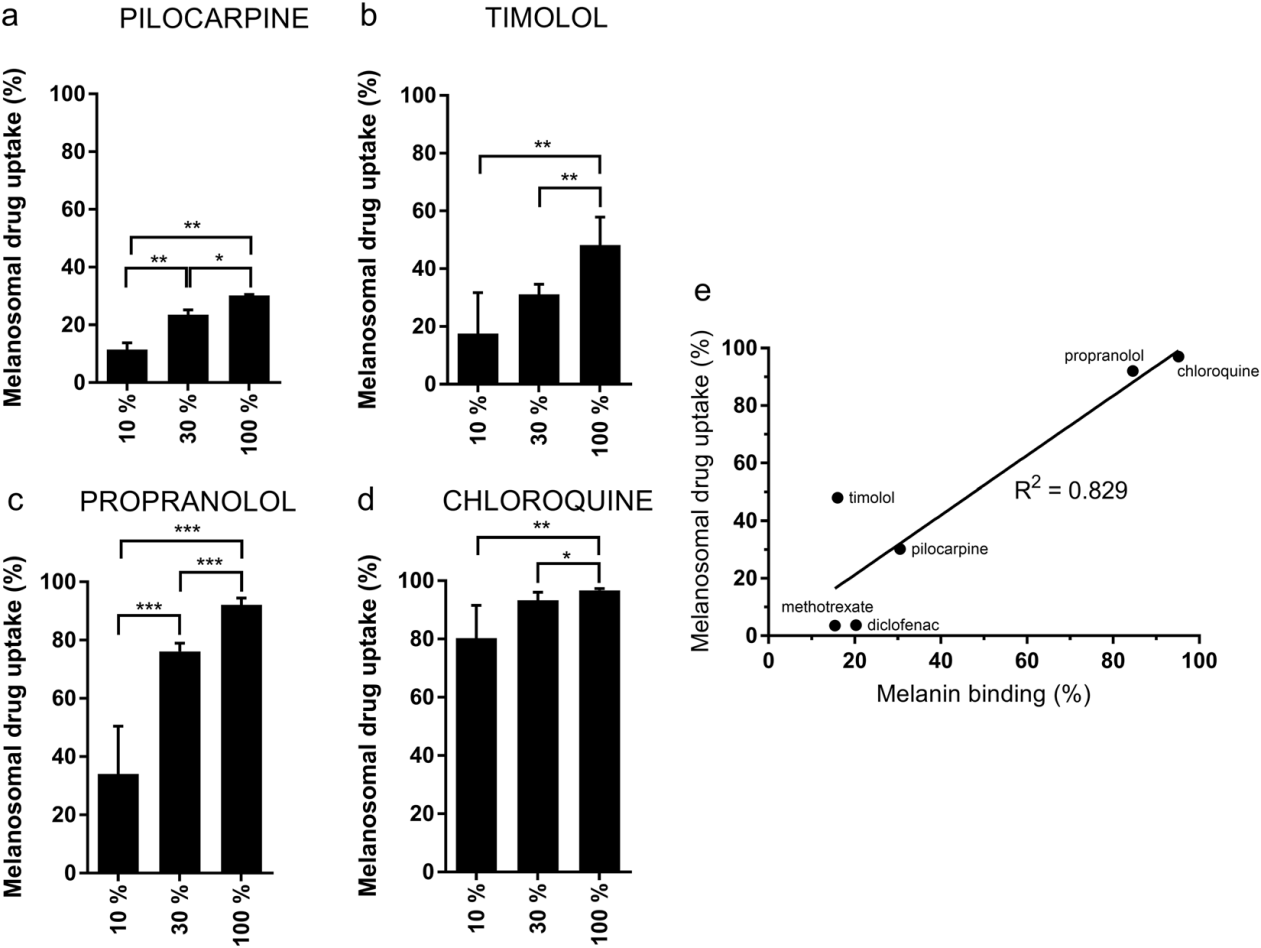
High melanin-binders propranolol (**c**) and chloroquine (**d**) show high melanosomal uptake in the ARPE-19mel cells whereas the amounts of low and intermediate melanin binders, pilocarpine (**a**) and timolol (**b**), in the melanosomes were modest. Melanosomal drug uptake was calculated as: melanosomal drug uptake (%) = ( 1 − (C_ARPE-19mel medium_/C_ARPE-19medium_)) * 100%. The drug uptake was studied with 2–6 replicates in each condition, and the bars display mean ± SD. Statistical significance (*p < 0.02, ** p < 0.005, ***p < 0.001) of the differences was determined using multiple t-tests with FDR approach (two-stage linear step-up procedure of Benjamini, Krieger and Yekutieli, with Q = 1%) without assuming a consistent SD (GraphPad Prism 7.04). (**e**) Melanosomal drug uptake in cellular environment correlates with *in vitro* melanin binding at pH 7.4. Melanosomal drug uptake in the cells with 100% melanosome levels was correlated with previously published melanin binding percentages *in vitro*. The data of methotrexate, diclofenac, pilocarpine, propranolol are from^15^, timolol and chloroquine results from^35^.

We studied uptake of drugs into melanosomes with ARPE-19mel cells displaying different levels of pigmentation. Low melanin-binder drug (pilocarpine) and intermediate melanin-binder (timolol) showed lower melanosomal uptake than the high melanin-binders (propranolol, chloroquine) (Fig. 4a–d). Methotrexate and diclofenac are low melanin binders that showed very low drug uptake into melanosomes (<5%) (Supplementary material).

There was clear correlation between cellular melanin content and cellular drug uptake (Fig. 4) and differences in drug uptake were significant (for statistics, see Supplementary material). Cellular uptake of drugs correlated with previously reported *in vitro* melanin binding values (R^2^ = 0.829)^15,35^ (Fig. 4e).

## Discussion

In this study, isolated melanosomes from porcine RPE were administered onto human ARPE-19 cell culture to re-pigment the cells. The ARPE-19 cells were able to internalize melanosomes at levels corresponding up to 1180 pg melanin/cell, an amount that is close to the melanin levels in the isolated porcine RPE cells (1110 pg melanin/cell, Fig. 2a). At higher melanosome doses, even 3670 pg melanin/cell was reached in the ARPE-19mel cells (Fig. 2a). Thus, phagocytosis-based feeding of melanosomes into the ARPE-19 cells was effective, resulting in even 3.3-fold higher levels of pigmentation than in the intact porcine RPE. High melanosome levels are understandable, since phagocytosis is effective internalization mechanism and cellular melanosome turnover is negligible. Melanosomes seem to be stably stored in the cytoplasm of the cells (Supplementary material), and not subjected to degradative processes. In our laboratory, the melanosomes in the ARPE-19mel cells retained for at least 4 weeks, when the melanosomes were inserted into confluent cell cultures (unpublished observations). This finding is similar with the earlier literature describing the artificial re-pigmentation of human RPE cultures with isolated melanin^33^. Consequently, we do not anticipate significant decrease in the cellular pigment content during continuous cultivation.

We calculated the theoretical melanin content in the human RPE based on the observed melanin content in the porcine RPE cell (1110 pg/cell) and the previously reported cell number in the human RPE (reported range 2 130 500 to 4 653 200 cells per RPE^36^, Supplementary Table S1). This calculation corresponded to 2.3–5.1 mg of melanin in the human RPE (average 3.9 mg). The total melanin content in the human RPE has not been reported previously, but we believe that our estimation is reliable. Previously reported melanin amount in the human RPE-choroid was 6.7 mg for individuals with light-colored irides and 8.4 mg for brown-eyed individuals^37^. The choroid may contain more melanin than the RPE and the inter-individual variation in the RPE melanin content in normal eyes is low^38^, but the melanin levels decrease with aging^38,39^.

Our melanosome isolation method produces intact, functional and pure fraction of melanosomes with proven activity of ATPases^34^. In this study, we characterized the melanosomal membrane of the inserted melanosomes inside the cells by overexpressing GFP-Rab27a vector in ARPE-19mel cells. We showed that Rab27a, a melanosomal membrane protein involved in organelle motility^40–43^, was sorted to the melanosomal membranes (Fig. 3c) indicating that the transferred exogenous melanosomes have functional melanosomal membranes. Intact melanosome membrane is crucial for the functions of the melanosomes, because several proteins responsible for ion exchange, melanosomal pH control, and transport of tyrosine are located in the melanosome membranes of the RPE^44^. Furthermore, melanosomal membrane has impact on drug distribution from the melanosomes into the cytosol, thereby affecting the cellular retention of drugs in pigmented cells^16^.

Extensive amount of melanin in the RPE^45^ contributes also to drug accumulation into the cells^18,20^. Drug-melanin interactions play an essential role in ocular pharmacokinetics and toxicity, but as reviewed earlier, drug-melanin interactions have been investigated mostly with isolated or synthetic melanin or isolated melanosomes^16^. Such studies inform about the extent and equilibria of melanin binding, but not about the kinetics at cellular level. Therefore, the literature describes drug-melanin interactions without considering organellar or cellular environments. Some recent studies have compared the drug uptake into pigmented (cultured porcine RPE cells) and non-pigmented (ARPE-19) RPE cells^35,46^. The compounds with high affinity to melanin had much greater cellular accumulation in pigmented than non-pigmented RPE cells (K_p_ values even 10^3^–10^4^)^46^. Previous simulations also suggest that melanin content is a critical factor that controls cellular kinetics in pigmented cells^16^. The pigment levels in primary RPE cultures decrease rapidly during cell duplication and this factor cannot be controlled or prevented. Therefore, control of the melanin content in the cells would be an important experimental asset, especially for mimicking the changes of pigmentation in the diseased and aging eyes^9,38,39^.

We quantified melanosomal drug uptake by considering drug accumulation into other cell components and culture vessel as background (Eq. 1). Furthermore, other relevant factors, such as active influx or efflux by transporters and metabolic activity, are considered to be identical in ARPE-19 and ARPE-19mel cells. This approach is supported by the earlier reports that showed that the pigment binding does not affect the ratio of unbound drug inside and outside of the cells (i.e. intracellular bioavailability, F_ic_)^46^.

Clear differences in melanosomal drug uptake in the ARPE-19mel cells were seen among six drugs further demonstrating the usefulness of these cells. Accumulation into the melanosomes was a dominant factor in cellular uptake for high melanin-binders, since >90% of the administered compound (propranolol, chloroquine) resided in the melanosomes (Fig. 4c,d). In contrast, the melanosomal fraction of low melanin-binding drugs (methotrexate, diclofenac) was less than 5%. The modest binders (pilocarpine, timolol) had intermediate melanosomal uptake levels (Fig. 4). Correlation between *in vitro* melanin binding and the melanosomal drug uptake in the cells suggests that *in vitro* binding assays can predict melanin binding in the cells. Larger datasets are, however, needed to generate predictive models based on chemical structure and melanin binding.

This study proves that melanosome uptake into ARPE-19 cells can be controlled, enabling generation of RPE cells with controlled levels of pigmentation (Fig. 2a,b,d–f). The control of pigment content enables cellular level studies that quantitate equilibria and kinetics of drug binding to melanin. The model can also be used to investigate cellular pharmacokinetics in conditions that mimic decreased RPE pigmentation during disease and aging. For instance, loss of RPE melanin is associated with age-related macular degeneration^9^. Indeed, the level of pigmentation clearly affects the cellular drug levels (Fig. 4a–d), suggesting that ARPE-19mel cells can be used to adjust the melanosome content and mimic different clinical situations. Our cell model could be used as a tool in drug discovery when easy-to-use cells are needed in the drug screening programs. Importantly, the active transporter profile of ARPE-19 cells is similar with the differentiated human primary RPE cells^25^, supporting the validity of this cell model. ARPE-19mel cells can also be used to study drug retention in the melanosomes and profiles of free drug concentrations in the cells.

In conclusion, we described continuously growing and pigmented RPE cell model in which the level of pigmentation can be conveniently controlled at relevant levels of melanin. Our drug uptake assay links melanin binding with relevant cellular environment, providing an improved tool to assess the kinetic impact of pigment binding in the RPE cells. Re-pigmented ARPE-19mel cells can be used to model the clinical differences in the RPE pigmentation. Importantly, our cell model contains melanosomes with intact membrane crucial for melanosome function and pharmacokinetics. We believe that this cell model will be useful in ocular cell biology, pharmacology, toxicology and drug development.

## Methods

Overview of the study protocol is presented in Fig. 1.

### RPE cell isolation from porcine eyes

Porcine eyes were received from a local slaughterhouse. Extraocular tissues, anterior eye segment, vitreous and neural retina were removed with scalpel and tweezers. Phosphate-buffered saline (PBS) was added into the eye cup, and the RPE cells were detached by gentle scraping with small paintbrush. The cells were collected with a pipet and PBS was removed with centrifugation (6238 g, 5 min at +4 °C). The RPE cell pellets were stored at −20 °C before isolation of melanosomes or melanin.

### Quantitation of melanin content in freshly isolated porcine RPE cells

In order to measure melanin content in intact porcine RPE cells (pRPE), freshly isolated pRPE cells were washed by centrifugation, re-suspended into PBS and transferred onto 96-well plate. The melanin concentration was measured using absorbance at 595 nm (EnVision plate reader, PerkinElmer or Wallac Victor² 1420 Multilabel counter, Perkin Elmer, USA). Melanin standards (0.00, 0.05, 0.10, 0.25, 0.50, 1.00 and 2.00 µg melanin/µl) were generated from isolated porcine RPE melanin^15^. Melanin shows a very broad UV-visible absorbance spectrum^47^, enabling quantitation of porcine RPE melanin at wavelengths of 400–600 nm (Supplementary Fig. S1). Melanin quantitation with absorbance method has been shown to be consistent with melanin quantitation using HPLC^48^. After the melanin quantitation, the cells were counted with Bürker cell calculation chamber, and melanin amount/cell was calculated.

### Melanosome isolation

Intact melanosomes were isolated from porcine RPE as described earlier^34^ (see the original publication for detailed instructions). Briefly, the pRPE cell pellets were thawed on ice, suspended into hypotonic buffer (10 Mm Tris-HCl, 10 Mm NaCl, 1.5 mM MgCl_2_) and lysed with nitrogen cavitation after 15 min equilibration at 450 psi (Parr Instruments, chamber was kept on ice during the equilibration). Crude melanosomal fraction was pelleted with 3000 g centrifugation, re-suspended into 10 mM Tris-HCl, 150 mM KCl buffer (pH 7.40) and layered on top of discontinuous OptiPrep® gradient (bottom-top: 1.5 ml 50%; 1.5 ml 35%; 1 ml 30%; 1.5 ml 20%; 2.0 ml 15% OptiPrep® solutions). Melanosomal fraction was pelleted at 135 000 g centrifugation (SorwallTM WX Ultra Centrifuge, TH-641 Swinging Bucket rotor, Thermo Fischer Scientific), re-suspended into 10 mM Tris-HCl 150 mM KCl buffer (pH 7.40) and further purified with subsequent OptiPrep® gradient centrifugation. Resulting melanosomal pellet was re-suspended into MES buffer (25 mM MES, 5 mM NaCl, 115 mM KCl, 1.3 Mm, pH 7.40) and centrifuged at 10 000 g to remove any residual OptiPrep® solution. The washing step was conducted twice, and the melanosomal pellets were stored at −20 °C until further processing. Melanosomal melanin content was measured by the spectrophotometry method (A690 nm or 595 nm) as described above.

### Cell culture

Non-pigmented ARPE-19 human RPE cell line (CRL-2302, ATCC) was maintained in DMEM/12 medium (Gibco BRL 31330-038). The medium was supplemented with 10% FBS, 2 mM L-glutamine, 100 U/ml penicillin and 100 µg/ml streptomycin. The cells were grown at +37 °C and 5% CO_2_.

### Re-pigmentation of ARPE-19 cells

Melanosome containing ARPE-19mel cells were generated as described below. On day one, ARPE-19 cells were seeded on 96-well plate (20 000 cells/well, equals to 62 500 cells/cm^2^). Approximately 24 hours after seeding, different amounts of melanosomes (0, 2.53, 7.58, 22.73, 68.08 and 204 µg of melanin per well) were applied onto the culture wells as triplicates. Thereafter, the cells were cultured for 6–7 days. After melanosome administration, medium was gently renewed once before the analysis, carefully avoiding removal of non-phagocytosed melanosomes. In addition to single dose, we tested also repeated delivery of melanosomes thrice (3 × 68 µg melanin per well) at intervals of four days.

### Quantitation of melanin content in ARPE-19mel cells

Six to seven days after melanosome administration, cells were washed three times with culture medium to remove non-phagocytosed melanosomes. After washing, the growth medium was replaced with PBS and melanin content in the ARPE-19mel cells was quantitated with the spectrophotometric method as described above for isolated porcine RPE cells. The standards of porcine RPE melanin were added to the non-pigmented ARPE-19 control cells to ensure identical background in the standards and test samples. After melanin quantitation, the cells were trypsinized and the number of cells per well was counted. Melanin content was normalized to the cell number, resulting in melanin amounts per cell. The same procedure was done with intact porcine RPE cells (see above).

### Transmission electron microscopy

Two weeks after melanosome dosing, the cells were fixed and electron microscopic examination was carried out as described earlier^34^.

### Transfection of ARPE-19mel cells with GFP-Rab27a expression vector

Localization of melanosomal Rab27a protein was tested by transfecting ARPE-19mel cells with expression vector (GFP-Rab27a) for Rab27a protein^43^ that is fused with green fluorescent protein (GFP). For transfection, ARPE-19 cells were seeded on Ibidi µ-Slide (80826, Ibidi) (60 000 cells/cm^2^). On the following day, single dose of melanosomes (corresponding to 10 µg melanin/well) was given to the cells. Five days later ARPE-19mel cells were transfected with GFP-Rab27a plasmid (0.5 µg DNA/well) by using Lipofectamine2000 (P/N100014469 Invitrogen) as a carrier according to manufacturer´s instructions. Two days after transfection, expression of the GFP reporter in the cells was imaged by using Zeiss LSM800 Airyscan confocal microscope.

### Drug uptake studies

Uptake of six different drugs (Table 1) was studied in non-pigmented ARPE-19 cells and pigmented ARPE-19mel cells. The melanosome administration was conducted as described above using three melanosome doses (corresponding to 7 µg, 20 µg and 68 µg of melanin). These melanosome doses correspond to 10, 30 and 100% levels of pigmentation. The highest external melanosome dose (68 µg) results in similar cellular pigmentation with the native pRPE (i.e. normalized as 100%).

**Table 1:** Model compounds and their internal standards and their selected physicochemical properties.

Compound	pK_a_, most acidic	pK_a_, most basic	logD_7.4_ (predicted)	Manufacturer	Internal standard	Manufacturer
pilocarpine	—	7.02	−0.4	Santen Oy	atenolol-d7	Sigma Aldrich
diclofenac	—	4.2	1.4	Sigma Aldrich	diclofenac-d4	Toronto Research Chemicals
methotrexate	3.5	5.6	−5.1	Fluka	methotrexate-d3	Sigma Aldrich
timolol	—	9.4	−0.6	MP Biomedicals	rac-timololi-d5 maleate	MP Biomedicals
propranolol	13.8	9.5	0.8	Sigma Aldrich	rac-propranolol-d7	Toronto Research Chemicals
chloroquine	10.4	—	1.6	Sigma Aldrich	chloroquine-d4-phosphate	Sigma Aldrich

pK_a_ and predicted logD_7.4_ values collected from earlier literature^46,49^, descriptors originally obtained with ACDLabs® Software.

Drug uptake study was initiated 4–5 days after melanosome feeding. The experiments were conducted in 2–6 replicate wells, separately for each compound. The cells were exposed to 4 µM of each drug for 20 hours in serum-free culture medium. Then, samples from the medium samples were collected and diluted 2:5 into acetonitrile (ACN) with 0.2% formic acid. The samples were centrifuged at 15 000 g for 5 min, and the supernatant was collected and diluted 1:10 into mixture of ACN-water (60:40). Finally, internal standards were added at final concentration of 100 nM. Different internal standards were used for each compound (Table 1). The melanin amounts in the wells were measured after the uptake experiment (see above).

### LC-MS/MS

Drugs were quantitated with liquid chromatography mass spectrometry (LC-MS) analyses that were carried out with Acquity i-class UPLC (Waters, Massachusetts, USA) coupled with Xevo TQ-S triple quadrupole mass spectrometry (Waters). Methods were based on previous studies^46,49^, with minor modifications. Chromatographic separation for all six compounds was carried out using Waters HSS-T3 column (2.1 × 100 mm, 1.8 µm) and gradient run. The mobile phases were prepared as binary phases. Phase A was purified water with 0.1% of formic acid and phase B contained 100% lc-ms grade acetonitrile (Chromasolv^TM^). For pilocarpine and methotrexate, the percentage of organic solvent in the gradient solution shifted from 10% to 100% during 3 minutes. Total run time was 9 minutes including column flush and stabilization to the initial state. For chloroquine, timolol, propranolol and diclofenac, the gradients were similar, but flow rate of 0.4 ml/min was used. Injection volumes were 0.25 µl for timolol, 1 µl for propranolol, chloroquine, methotrexate and pilocarpine and 1.5 µl for diclofenac. Flow through needle system was used. Column temperature was for methotrexate and pilocarpine 34 °C and for others 26 °C while sample tray temperature was kept at +10 °C for all samples.

Electrospray ionization on positive mode was used in the analyses. Desolvation temperature was 500 °C and desolvation gas flow 1000 l/h for chloroquine and timolol, whereas settings of 600 °C and 900 l/h, respectively, were used for the other analytes. Other parameters were identical for all compounds: source temperature 150 °C, cone gas flow 150 l/h, nebuliser gas flow 7 bar, collision gas flow 0.15 ml/min. The only exception was the collision gas flow for chloroquine (0.25 ml/min). Nitrogen was used as a desolvation gas (Aga, Helsinki, Finland) and argon as a collision gas (Aga). Capillary and cone voltages, collision energy and dwell time were tuned individually for each compound (Table 2). To reach better selectivity we selected two product ions for each compound (Table 2) and multiple reaction monitoring (MRM) was used for data recording. Minimum of eight calibration curve levels were used for quantification while quality control (QC) samples used to monitor precision and bias of the assay. Data were processed with MassLynx V4.1 software (Waters).

**Table 2:** Mass spectrometry parameters and analyzed ions.

Compound	Precursor ion (m/z)	Product ion (m/z)	Capillary voltage (kV)	Cone Voltage (V)	Collision energy (V)	Linearity range (nM)	Dwell time (s)	Retention time (min)
chloroquine	320.1	142.1	1.2	7	22	1–1000	0.070	1.17
	320.1	246.9	1.2	7	20		0.070	1.17
chloroquine-d4	324.0	251.1	1.2	7	22		0.070	1.17
	324.0	146.1	1.2	7	24		0.070	1.17
timolol	316.9	261.0	3.2	4	16	0.5–750	0.068	1.47
	316.9	73.9	3.2	4	22		0.068	1.47
timolol-d5	322.1	266.1	3.2	2	17		0.068	1.47
	322.1	79.1	3.2	2	22		0.068	1.47
methotrexate	454.9	308.0	3.5	2	20	1–750	0.053	1.52
	454.9	175.8	3.5	2	42		0.053	1.52
methotrexate-d3	457.9	311.0	3.5	2	20		0.053	1.52
	457.9	175.8	3.5	2	42		0.053	1.52
pilocarpine	209.0	120.9	3.5	2	25	0.5–1000	0.053	1.40
	209.0	95.0	3.5	2	27		0.053	1.40
atenolol (IS for pilocarpine)	267.1	145.0	3.5	2	25		0.082	1.36
	267.1	190.0	3.5	2	20		0.082	1.36
propranolol	260.1	116.1	3.5	2	14	0.5–1000	0.072	1.77
	260.1	183.0	3.5	2	16		0.072	1.77
propranolol-d7	267.2	123.2	3.5	2	18		0.072	1.77
	267.2	79.1	3.5	2	20		0.072	1.77
diclofenac	297.0	250.9	3.5	2	9	1–750	0.080	2.99
	297.0	214.9	3.5	2	41		0.080	2.99
diclofenac-d4	299.9	253.9	3.5	2	15		0.080	2.99
	299.9	219.0	3.5	2	20		0.080	2.99

### Calculation of melanosomal drug uptake

The melanosomal drug uptake was calculated using the Eq. 1 below:

1 $$Melanosomal\,drug\,uptake\,( \% )=(1-\frac{Cmedium\,ARPE-19mel}{Cmedium\,ARPE-19})\times 100\, \% $$

### Statistical testing

Statistical significance of the differences in the melanin content between the generated ARPE-19mel cells and freshly isolated porcine RPE cells were evaluated with unpaired t-tests without assuming a consistent standard deviation (SD). The p-values were determined with false discovery rate (FDR) approach using two-stage linear step-up procedure of Benjamini, Krieger and Yekutieli, with Q = 1%, using GraphPad Prism 7.04 software (Fig. 2a). The same statistical method was used to evaluate the influence of pigmentation on melanosomal drug uptake (Fig. 3a–d).

Correlation coefficients (Figs 2b, 3f) were obtained with linear regression using GraphPad Prism 7.04 software. Residual plots are displayed in the Supplementary material.

## Supplementary information

Supplementary Information: Characterization of artificially re-pigmented ARPE-19 retinal pigment epithelial cell model
