# Supplementary material for: Characterization of artificially re-pigmented ARPE-19 retinal pigment epithelial cell model
Source: Sci Rep. 2019 Sep 24;9:13761. doi: 10.1038/s41598-019-50324-8 (PMC6760193; doi:10.1038/s41598-019-50324-8)
Supplement: Supplementary file 1 — Supplementary Information: Characterization of artificially re-pigmented ARPE-19 retinal pigment epithelial cell model [file 41598_2019_50324_MOESM1_ESM.pdf]

Supplementary Information

Characterization of artificially re-pigmented ARPE-19 retinal pigment epithelial cell model

Laura Hellinen[1], Marja Hagström[2], Heidi Knuutila[1], Marika Ruponen[1], Arto Urtti[1, 2, 3], Mika Reinisalo[1, 4]
[1]School of Pharmacy, Faculty of Health Sciences, University of Eastern Finland, 70210 Kuopio, Finland
[2]Drug Research Programme, Division of Pharmaceutical Biosciences, Faculty of Pharmacy, University of Helsinki, P.O. Box 56, FI-00014 Helsinki, Finland
[3]Laboratory of Biohybrid Technologies, Institute of Chemistry, St. Petersburg State University, Peterhoff, 198504 St. Petersburg, Russian Federation
[4]Institute of Clinical Medicine, Department of Ophthalmology, Faculty of Health Sciences, University of Eastern Finland, 70210 Kuopio, Finland

Feasibility of the absorbance method to measure pigment content

We tested the feasibility of broad range of wavelengths by scanning absorbance (abs 375-625 nm) of melanin dilutions 0.0-1.0 µg/µl isolated from the porcine eye. Figure S1 shows that absorbance values of different melanin dilutions decrease only slightly at higher wavelengths enabling reliable melanin quantitation at visible wavelengths. The absorbance was measured by using a Envision plate reader (Perkin-Elmer). We chose the wavelength of 595 nm, and further tested the linearity of measured absorbance with increasing melanin concentrations. The Figure S2 below shows, that the increase in absorbance was linear. In our measurements, the absorbance method resulted in 80-120 % accuracy, when the standard sample concentrations were calculated with the standard equation verifying that the method and chosen wavelength correctly predicted the melanin concentration.

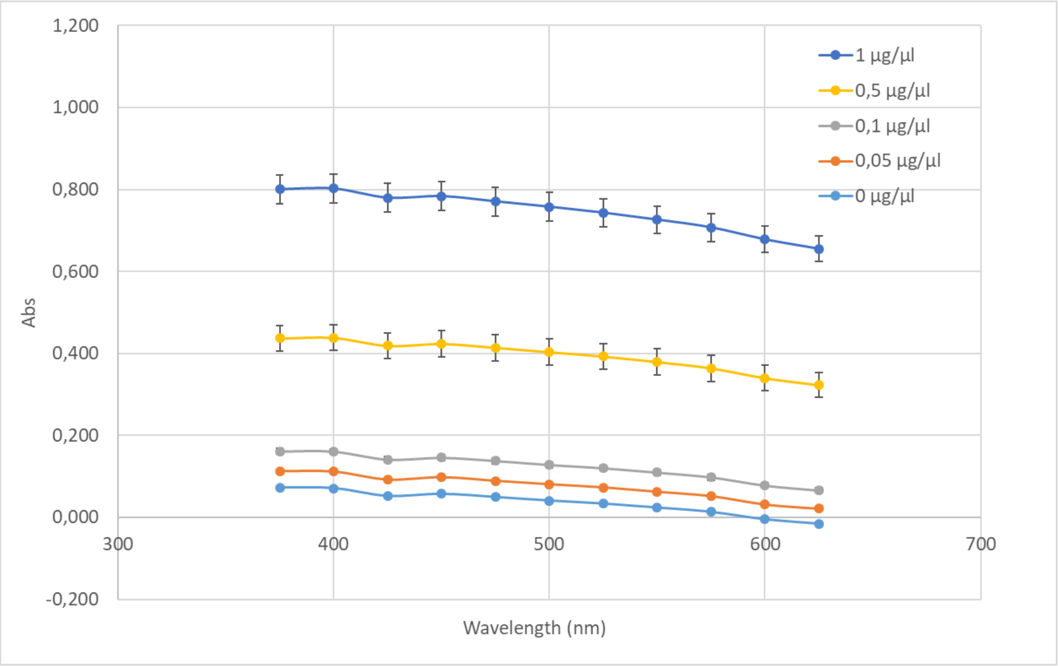

Figure S1. Porcine eye melanin absorption scanning 375-625 nm. Data are expressed as the mean ± sd (n=3).

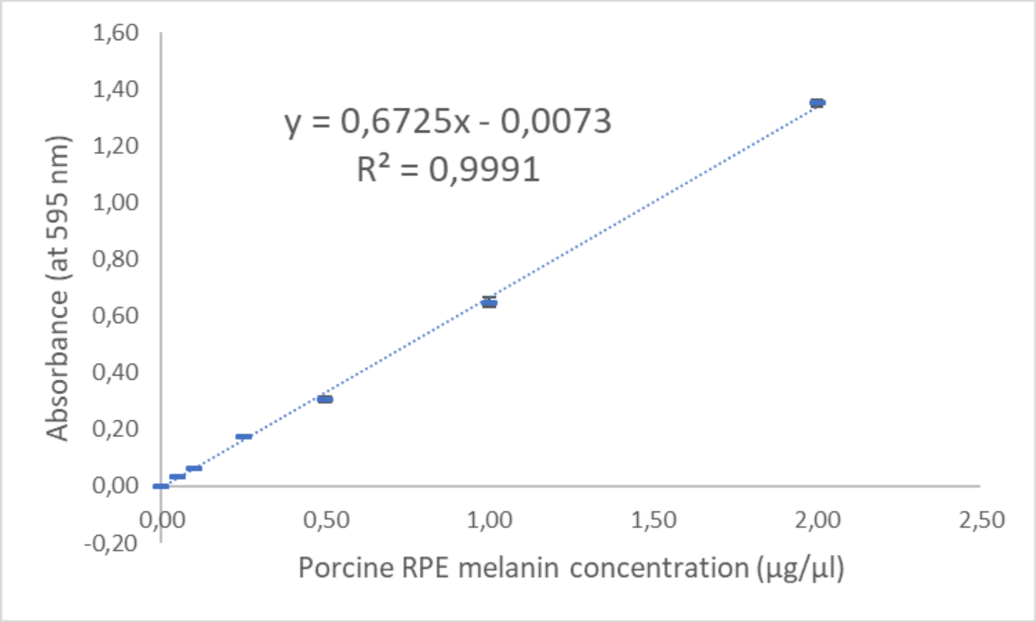

Figure S2. Standard curve for porcine RPE melanin standard samples (0-200 µg; 0-2 µg/µl) based on absorbances at at 595 nm. Each data point is the mean ± SD of 3 replicate samples. The standard deviations are small (0.001-0.017).

<u>Pigment content and melanosomal drug uptake</u>

Supplemental Table 1 describes the measured pigment content in the intact porcine RPE cells and different ARPE-19mel cells. The pigment content was determined with a spectrophotometer (see materials and methods). The pigment recovery (%) was determined by comparing the applied pigment doses in the well and measured amounts of cellular pigment (Equation S1).

$$\text{Pigment recovery }(\%) = \frac{\text{Measured pigment amount }(\mu\text{g/well})}{\text{Inserted pigment dose }(\mu\text{g/well})} \times 100\ \% \qquad \text{Eq. S1}$$

The melanin dose of 68 µg resulted in similar pigmentation with normal porcine RPE cells. Moreover, the recovery with each pigment dose was close to 100 % indicating high yield in pigment delivery and stable retention of the internalized melanosomes. Comparison of the pigment content/cell to melanosome dose resulted in strong correlation (R^2 = 0.9988, Fig. 1b, main article) verifying that the resulting pigment content can be adjusted with the dose of melanosomes. Table S1 includes also an estimation of the pigment content in the human RPE based on previously reported RPE cell counts [1] and the pigment content in the intact porcine RPE determined in this study (Table S1).

The amount of melanin was measured after the drug uptake experiments, and the values are presented in the Supplementary Table 2. The 100 % stage of pigmentation represents the ARPE-19mel cells generated by dosing the cells

melanosomes with 68 µg melanin, as its pigmentation was similar to the levels in the porcine RPE (Table S1). The administered and measured amounts of pigment are displayed in Figure S3.

The detailed melanosomal drug uptake data is presented in the Supplementary Table S2.

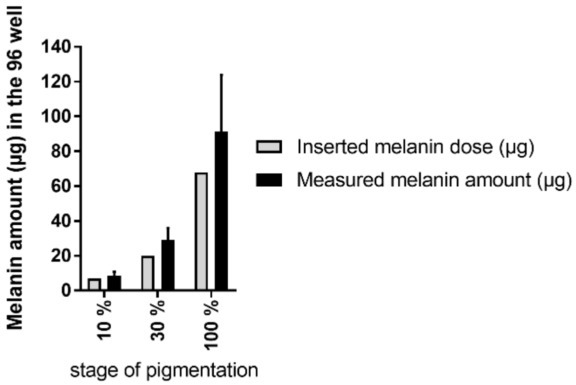

Figure S3. Pigmentation in the ARPE-19mel cells**.** The inserted melanin doses were 7 µg for 10 %, 20 µg for 30 % and 68 µg for 100 % level of pigmentation. The melanin amounts measured after the uptake experiments displayed similar levels of pigment as inserted into the cells.

Supplemental Table S1. Pigment content in porcine RPE cells, ARPE-19mel cells and estimation of the human RPE pigment content

ARPE-19mel cells, inserted pigment dose (µg melanin/well)	ARPE-19mel cells, pigment dose per surface area (µg melanin/cm^2)	Theoretical pigment dose per cell (pg melanin/cell)[a]	Measured pigment amount per well (µg melanin/well)	n	SD	Measured pigment amount inside the cells (pg melanin/cell)	n	SD	Pigment recovery (%)	Pigment recovery range (%)
**2.5**	8	125	3.8	6	0.3	67.4	6	20	152.1	142-180
**7.6**	24	380	9.5	6	1.4	156.2	6	27	125.3	111-163
**22.7**	71	1135	22.3	6	2.5	306.5	6	71	98.4	84-118
**68**	213	3400	69.1	9	13.7	1182	9	296	101.6	63-127
**204**	638	10200	176.7	9	30.1	3668	9	1106	86.6	67-108
**pRPE cells**	n.a	n.a	n.a	n.a	n.a	1110	8	268	n.a	n.a
**Estimation of the pigment content in human RPE**										
**Cell number**[b]										
**Range**	2 130 500 to 4 653 200 cells									
**Average ± SD**	3 556 290 ± 490 700 cells									
**Melanin amount in the entire RPE**										
**Range**	2.4 – 5.1 mg									
**Average**	3.9 mg									

[a]Calculation assumes no cell duplication after cell seeding at 62 500 cells/cm^2 (20 000 cells/well). The surface area of 96 well plate is 0.32 cm^2. [b]Previously reported by Panda-Jonas et al. 1996 [1]. Calculations based on the reported human RPE cell number x 1110 pg melanin/cell.

Supplemental Table S2. Melanosomal drug uptake and melanin amounts in the corresponding experiments

	10 % stage of pigmentation						30 % stage of pigmentation						100 % stage of pigmentation					
	Melanosomal drug uptake (%)			Melanin amount in the well (µg)			Melanosomal drug uptake (%)			Melanin amount in the well (µg)			Melanosomal drug uptake (%)			Melanin amount in the well (µg)		
**Compound**	**Average**	**SD**	**n**	**Average**	**SD**	**n**	**Average**	**SD**	**n**	**Average**	**SD**	**n**	**Average**	**SD**	**n**	**Average**	**SD**	**n**
**diclofenac**[a]	0.8	1.8	6	7.64	0.21	6	1.2	1.8	6	36.2	1.1	6	3.7	4.5	6	135.6	0.3	6
**methotrexate**[a]	5.1	3.4	6	6.60	0.01	6	3.9	4.1	6	33.3	2.0		3.5	5.2	6	100.4	12.2	6
**pilocarpine**	11.5	2.4	3	n.d			23.5	1.7	3	n.d			30.2	0.4	2	n.d		
**timolol**	17.6	14.2	6	10.6	4.03	6	31.2	3.5	6	23.3	4.8	6	48.2	9.7	6	57.5	1.1	6
**propranolol**	34.1	16.4	6	9.8	2.79	6	76.1	2.8	6	21.3	2.9	6	92.2	2.3	6	77.9	13.8	6
**chloroquine**	80.3	11.2	6	7.8	0.5	6	93.3	2.7	5	33.7	2.3	6	96.7	0.6	6	127.9	21.7	6
**average melanin amount/well (µg)**[b]				9.0	1.29	30				29.5	6.1	30				99.8	29.5	30

[a]Some cell wells in low-binder experiments (with diclofenac and methotrexate) resulted in negative values (drug concentrations higher in the ARPE-19mel than ARPE19 extracellular medium). These were considered as 0 % melanosomal drug uptake.

[b]Represents the average and SD values of the measured melanin amounts inside the wells after all the uptake assays conducted with the same pigmentation stage (all compounds included, n=30).

Statistical analysis

Statistical analyses were conducted as described in the main article. The Supplemental Table S3 describes the exact p-values that were used to assess the statistical significance of the pigment content in the cells for the melanosomal drug uptake. The Figure S4 shows the residual plots of the linear regression analysis.

Supplemental Table S3. Statistical tests of the drug uptake data: effect of pigmentation level on melanosomal drug uptake.[a]

**Compound**	**P-values**		
	**[b]10 % compared to 30 %**	**[b]30 % compared to 100 %**	**[b]10 % compared to 100 %**
**pilocarpine**	0.0020	0.014	0.0018
**timolol**	0.0456	0.0022	0.0014
**propranolol**	0.00010	0.00000080	0.0000062
**chloroquine**	0.0331	0.014	0.0050

[a]Statistical testing of the significance different stages of pigmentation and melanosomal drug uptake was conducted using un-paired t-test with FDR approach (two-stage linear step-up procedure of Benjamini, Krieger and Yekutieli, with Q = 1%) without assuming a consistent SD. Numbers of replicates of each condition are presented in the Supplemental Table 2 above.

[b]Stage of pigmentation: correspond to inserted melanin amounts of 7 µg for 10 %, 20 µg for 30 % and 68 µg for 100 % stage of pigmentation.

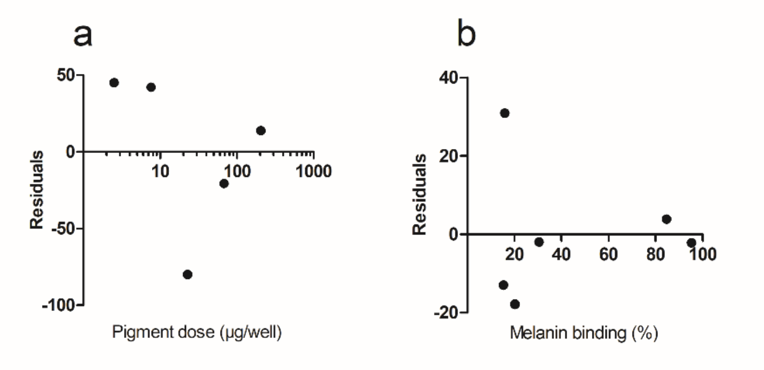

Figure S4. Plots describe the residuals from the linear regression analysis. (a) Residual plot of linear regression between pigment dose and observed melanin content inside the cells. **(b)** Residual plot of linear regression between melanosomal drug uptake and previously published in vitro melanin binding.

References

1. Panda-Jonas, S., Jonas, J. B. & Jakobczyk-Zmija, M. Retinal pigment epithelial cell count, distribution, and correlations in normal human eyes. *Am. J. Ophthalmol.* **121**, 181-189 (1996).
